# Immunoglobulin G Concentrations in Alpaca Colostrum during the First Four Days after Parturition

**DOI:** 10.3390/ani12020167

**Published:** 2022-01-11

**Authors:** Maria Mößler, Kathrin Rychli, Volker Michael Reichmann, Thiemo Albert, Thomas Wittek

**Affiliations:** 1 University Clinic for Ruminants, Department for Farm Animals and Veterinary Public Health, University of Veterinary Medicine Vienna, Veterinärplatz 1, 1210 Vienna, Austria; Thomas.Wittek@vetmeduni.ac.at; 2 Department for Farm Animals and Veterinary Public Health, Institute of Food Safety, Food Technology and Veterinary Public Health, University of Veterinary Medicine Vienna, Veterinärplatz 1, 1210 Vienna, Austria; Kathrin.Rychli@vetmeduni.ac.at; 3 TierMed Krieglach OG, Roseggerstraße 134, 8670 Krieglach, Austria; michael@reichmann.vet; 4 Institute of Food Hygiene, Veterinary Faculty at Leipzig University, An den Tierkliniken 1, 04103 Leipzig, Germany; albert@vetmed.uni-leipzig.de

**Keywords:** alpaca, colostrum, immunoglobulin G

## Abstract

**Simple Summary:**

During the first days after parturition, mammalian milk (colostrum) is specifically formulated to nourish newborns. Immunoglobulins are a particularly important component for newborn New World camelids, as their immune system is almost totally dependent on the intestinal transfer of colostral immunoglobulins to acquire passive immunity. In this study, colostrum samples were collected from 20 alpaca mares in the first four days after parturition and analyzed for their immunoglobulin concentration. Sampling started on the day of parturition. The associations of immunoglobulins with other components were determined. The immunoglobulin G (IgG) concentrations decreased significantly within the first four days after parturition. The correlation coefficients between IgG content and the content of various minerals were significant but variable. The correlation between IgG content and fat and lactose content was negative but between IgG content and protein content was highly positive. This strong association could be used for a brief estimation of the IgG content of the colostrum based on the measured protein concentration. The results of the present study can be used for the development of colostrum replacers where motherless rearing is required.

**Abstract:**

Colostrum provides the newborn with nutrients and immunoglobulins. Immunoglobulins and their intestinal transfer play a major role in the immune system of neonates since they are born agammaglobulinemic. In this study immunoglobulin G (IgG) content was determined in alpaca colostrum and the correlations of the IgG concentration by fat, protein, lactose and minerals were calculated. Colostrum samples were collected daily from 20 multiparous alpaca mares during the first four days after parturition. The IgG concentrations were determined by radial immunodiffusion using a Camelid IgG Test Kit. The IgG concentration decreased significantly from 26,319 mg/dL on day 1 to 3848.8 mg/dL on day 4. There were significant correlations between IgG concentration and the other components of the colostrum. While the correlations between IgG and fat (r = −0.69, *p* ≤ 0.001) and lactose (r = −0.64, *p* ≤ 0.001) were negative, the correlations with protein (r = 0.91, *p* ≤ 0.001), magnesium (r = 0.86, *p* ≤ 0.001) and cobalt (r = 0.87, *p* ≤ 0.001) were strongly positive. Due to the strong association, the colostrum protein concentration could be used for a brief estimation of the IgG content.

## 1. Introduction

Colostrum is the milk of mammals during the first days after parturition, which differs in composition from milk during lactation and plays an important role in the acquisition of passive immunity, development and survival of the newborn [1,2]. The major quality determining components of colostrum are immunoglobulins [3,4]. Due to the epitheliochorial placenta of alpacas crias are born agammaglobulinemic. Therefore, they should consume high-quality colostrum to gain passive humoral protection from their dams via intestinal absorption to be protected against infectious diseases [5,6,7,8,9,10]. If the dams do not provide a sufficient amount and quality of colostrum or even die during parturition efficient colostrum replacers are necessary. The formulation of colostrum replacers requires detailed knowledge of the composition of the colostrum of alpacas. However, the composition of alpaca colostrum and that of South American Camels (SAC) in general has been sparsely investigated [11,12].

One study examined the IgG content of colostrum collected from 25 llamas kept in Argentina prior to crias suckling [13]. Additionally, in Argentina 15 llamas were milked within the first 24 h after parturition and the samples were analyzed for their IgG content [14]. Flodr et al. [15] analyzed 26 alpaca colostrum samples collected in Peru immediately after parturition before the first suckling of the crias. In another study, colostrum samples were analyzed from 14 alpacas kept in the USA sampled immediately after parturition and 24 h later [16]. Bravo et al. [17] collected samples from 15 llamas and 15 alpacas kept in Peru before, immediately at and after parturition. These studies measured the IgG concentrations only at one defined time point after parturition. To our best knowledge, the changes of IgG in colostrum over time have not been investigated yet.

The major objective of this study was to determine the concentrations of IgG in the colostrum of alpacas over the first days after parturition to obtain data for the development of suitable replacements for motherless reared crias or for crias of agalactic dams. We further investigated the relationship of IgG concentration with other colostral components in particular fat, protein, lactose, and minerals and compared with those of other animal species. In cattle, goats, sheep and other mammals, the term colostrum refers to the first five days after parturition before the milk changes into mature milk [1,2,3,4]. We have adopted this period for alpacas, and therefore, the first four days were chosen.

We hypothesized (i) that the concentrations of IgG in colostrum change significantly within the first days after parturition, (ii) that there are associations between IgG and other components of the colostrum.

## 2. Materials and Methods

### 2.1. Animals and Sampling Procedures

The project was approved by the institutional ethics and animal welfare committee in accordance with GSP guidelines and national legislation (Ethic Code ETK-21/11/2016).

During the period from June to September 2017 samples were obtained at three alpaca farms with similar environmental, husbandry and feeding conditions located in close proximity in the Austrian federal state of Styria, district of Bruck-Mürzzuschlag. During this time, the animals grazed on a pasture at 700 to 1100 m above sea level and hay ad libitum was offered additionally. Colostrum samples were collected from 20 multiparous alpacas (Huacaya breed) over four days after parturition (farm A five mares, farm B seven mares, farm C eight mares). The teats were taped two hours before sampling to obtain enough colostrum while the crias were not separated and could always nurse between samplings. During sampling, the animals were fixed by the owner using a halter. The first sample was collected on the day of birth between two and four hours after parturition, which is referred to as “day 1”. The samples of the following days were taken every 24 ± 4 h.

### 2.2. Sample Preparation

The udders were palpated and visually examined conspicuous redness, swelling and induration to exclude animals with clinical mastitis. The teats were cleaned before sampling using a swab dipped in alcohol. All available colostrum was milked from all four udder quarters into sterile 15 mL tubes (Greiner Bio-One GmbH, Kremsmünster, Austria). The samples were aliquoted in Eppendorf^®^ Safe-Lock microcentrifuge tubes (volume 2.0 mL, Eppendorf Austria GmbH, Vienna, Austria) frozen without preservatives immediately afterward and stored at −18 °C until further examination. 

### 2.3. Sample Analysis 

IgG concentrations were determined by a radial immunodiffusion test (RID) using the Camelid IgG Test Kit (Triple J Farms, Bellingham, WA, USA) in duplicates. The analysis has been conducted according to the instructions provided by the manufacturers. Colostrum samples were thawed at room temperature and diluted in saline (1:10–1:40, depending on sample viscosity). Sample viscosity was visually estimated and the sample was diluted accordingly. Sample dilution was adjusted and IgG determination was repeated if necessary. Three standardized concentrations (203 mg/dL, 1452 mg/dL, 2851 mg/dL) were used on each 24-well plate. The remaining wells were filled with 5 µL of the diluted samples. The plates were incubated in plastic bags at 23 °C for 24 h. After 24 h the precipitin circle diameters (in mm) were measured. Using the reference values, a linear regression equation was created, which was used to determine the sample results. 

The methods for determining fat, lactose, and protein content as well as mineral analysis are described in detail by Mößler et al. [12]. Briefly, fat content was analyzed with the gravimetric method according to Weibull-Berntrop [18], lactose content with the UV-lactose/D-galactose method (Roche Diagnostics^®^, Mannheim, Germany) and protein content with the Kjehldahl method [19]. Minerals were analyzed by inductive coupled plasma optical emission spectrometry (ICP-OES) using Vista-Pro device (Varian Inc., Palo Alto, CA, USA) and by inductive coupled plasma mass spectrometry (ICP-MS) device Aurora M90 (Bruker Daltonics, Bremen, Germany).

### 2.4. Statistical Analysis

The statistical evaluations and data presentations were performed with the statistics program R-Studio (product version: 1.1.463, RStudio Inc., Boston, MA, USA). Descriptive statistics (minimum, maximum, median, first/third quartile, arithmetic mean and standard deviation) were calculated. Further analyses were performed using log transformed data. The IgG content was compared over time separately using a mixed linear model (measurement repetition). The individual alpaca mare was considered as a random effect, while the day in milk, the farm and the lactation number were fixed effects. The Bonferroni test was applied as a posthoc test. The significance of the differences between the individual days was calculated with a significance level of *p* < 0.05. The correlations between the individual components were calculated using Pearson’s Rank correlation coefficient.

## 3. Results

From each alpaca mare (*n* = 20) four samples were collected with two exceptions: one mare died on the third day, and one showed clinical mastitis on the fourth day. In total 77 samples were collected. The volumes which could be obtained varied between 12 and 28 mL with a median of 20.8 mL. There were no differences in the obtainable volume from day 1 to day 4.

### Immunoglobulin G

**Figure 1:**
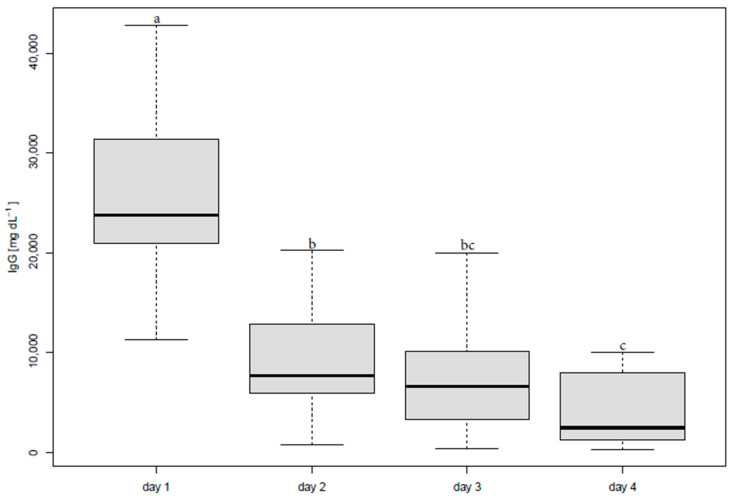
Boxplot of IgG concentrations in alpaca colostrum during four days after parturition, significant differences between the days are marked by different indices. Different letters indicate significant differences in values (*p* < 0.05).

The IgG content significantly decreased from day 1 (26,319 ± 8754.73 mg/dL) to day 2 (9234.4 ± 5778.96 mg/dL, Figure 1). Furthermore, there was a significant difference in the IgG concentration between day 3 (7280.1 ± 5014.32 mg/dL) and day 4 (3848.8 ± 3475.91 mg/dL).

**Figure 2:**
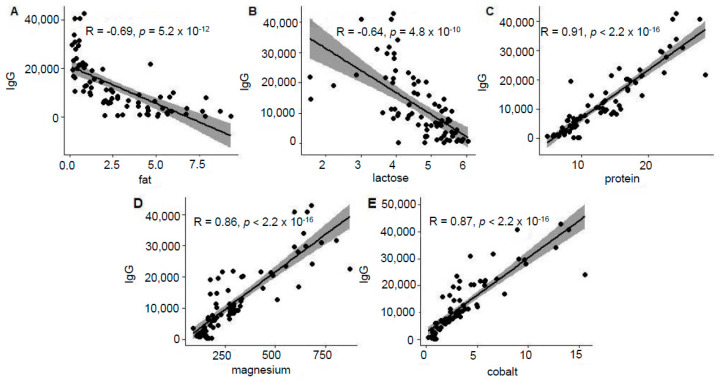
Associations between IgG and fat (**A**), lactose (**B**), protein (**C**), magnesium (**D**) and cobalt (**E**) concentrations in alpaca colostrum during four days after parturition.

We observed a negative association between IgG and fat (r = −0.69, *p* ≤ 0.001, Figure 2A) and lactose (r = −0.64, *p* ≤ 0.001, Figure 2B) content. IgG was positively correlated with protein content (r = 0.91, *p* ≤ 0.001, Figure 2C). The IgG content was also correlated with magnesium (r = 0.86, *p* ≤ 0.001, Figure 2D) and cobalt (r = 0.87, *p* ≤ 0.001, Figure 2E). The correlation coefficients between IgG content and the content of other minerals were lower but significant (Table 1).

## 4. Discussion

As our hypothesis suggested, the concentrations of IgG in the colostrum of alpacas significantly decreased within the first four days of lactation. The significant change in the IgG content from the day of parturition to the following day has also been described in other animal species, such as camels, cattle, goats and sheep [9,20,21,22]. The IgG content continued to decrease after the first 24 h after parturition, following the same progression as in other animal species.

In the present study, the colostrum on day 1 showed an IgG content of 26,319 ± 8755 mg/dL. In previous studies of alpaca colostrum IgG concentrations were found with 28,337 ± 5593 mg/dL [15] and 21,792 ± 786 mg/dL [17], respectively. Analyses of colostrum from llamas at birth showed IgG concentrations of 23,254.9 ± 7068 mg/dL [13] and 22,313 ± 3806 mg/dL [17].

Studies in which the alpaca colostrum was examined with a different test method namely enzyme-linked Immunosorbent Assay ELISA, obtained IgG content with 19,620 ± 2590 mg/dL [16] and 4254 ± 2779 mg/dL [14]. It has already been reported that results from the two test methods, RID and ELISA, are not directly comparable and that generally lower concentrations are measured with ELISA compared to RID [23,24,25,26,27]. 

Compared to our study the colostrum IgG concentrations of ruminant species analyzed with RID on the first day after parturition are substantially lower. Colostrum from cattle contains between 6360–9960 mg/dL [21,28,29,30,31,32,33], from sheep contains between 4810–6090 mg/dL [34,35,36] and from goat contains between 2151–7201 mg/dL [22,37,38,39] IgG.

Al-Busadah [40] tested the absorption abilities of newborn camels of IgG from colostrum from other species. The crias received either colostrum from their own dam or pooled colostrum with similar IgG content from cattle or goats. The study showed a similar absorption of IgG with goat and cattle colostrum feeding. Whether this also provides protection against animal-specific diseases remains to be researched. However, if no species-specific colostrum is available, then goat or bovine colostrum could be an option.

In this study, a significant correlation (r = 0.91, *p* ≤ 0.001) between the IgG and protein concentration was demonstrated. In bovine colostrum correlations of r = 0.86 [41] and r = 0.82 [42] between IgG content and protein content were found. Additionally, in goat colostrum the IgG concentration associated with the protein content (r = 0.9 [39], r = 0.7 [43]). In a different study using ELISA for the IgG determination, there was a weaker correlation between the IgG and the protein content in bovine colostrum (r = 0.66 [44]). 

On the other hand, a negative correlation between IgG and fat content (r = −0.69, *p* ≤ 0.001) was observed, while studies in goats reported a moderate or weak positive correlation between IgG content and fat content (r = 0.44 [39], r = 0.31 [43]). In cattle, only weak negative correlations between the IgG and fat content were demonstrated (r = −0.36 [41]). The negative correlation between the IgG and lactose content in this study (r = −0.64, *p* ≤ 0.001) has also been found in goat colostrum (r = −0.6 [43]). To our knowledge, correlations between IgG content and other components of the colostrum have not been reported in SAC. In our study, we found significant correlations between IgG content and two minerals (magnesium, cobalt). We are not aware that these associations have been described elsewhere for colostrum of other animal species and we are also not aware of their biological relevance.

Correlations studies of colostrum components were mostly conducted to test the use of rapid on-farm tools like colostrometer or refractometer for colostrum quality determination. RID is the reference method for IgG determination in colostrum, but it takes at least 24 h to obtain a result. Furthermore, it is an expensive method [45]. The colostrometer measures the specific gravity, which is influenced by the temperature and dry matter content of the colostrum. Higher solids and/or fat content result in a higher specific gravity value [46,47]. Digital or optical refractometers measure the refractive index of liquids on a Brix scale with acceptable sensitivity and specificity compared with RID and it is not sensible to temperature [30,45,48,49]. Flodr et al. [15] determined the IgG content of alpaca colostrum by refractometer and RID analysis and detected a strong correlation between the two methods. For the use of these on-farm tools in SAC, however, cut-off values for the evaluation of the result are not yet available and must first be determined. It would then be possible to check the quality of the colostrum quickly and inexpensively and, if necessary, promptly intervene and supplement adequately.

## 5. Conclusions

In conclusion, the concentrations of IgG in the colostrum of alpacas decrease significantly within the first four days after parturition. Compared to other animal species, the IgG concentration in alpaca colostrum is similar to those of other SACs, while substantially lower contents have been reported for bovine, caprine and ovine colostrum. The IgG content correlated negatively with the fat and lactose content, and positively with the protein content. Due to the strong correlation, a high colostrum protein concentration could be used as a predictor for a sufficient IgG content in the colostrum of alpaca.

## Figures and Tables

**Table 1:** Correlation coefficients and significance levels between IgG content and other mineral content in alpaca colostrum from 20 alpaca mares during four days after parturition (*n* = 77).

Correlation Partners	Correlation Coefficient (r)	Significance Level (*p*)
IgG/S	0.62	<0.001
IgG/Zn	0.53	<0.001
IgG/Ca	0.52	<0.001
IgG/Se	0.52	<0.001
IgG/Sr	0.46	<0.001
IgG/Ba	0.45	<0.001
IgG/P	0.45	<0.001
IgG/Fe	0.39	<0.001
IgG/Cu	−0.36	<0.01
IgG/U	−0.31	<0.01
IgG/Tl	0.29	<0.01

S: Sulfur, Zn: Zinc, Ca: Calcium, Se: Selenium, Sr: Strontium, Ba: Barium, P: Phosphorus, Fe: Iron, Cu: Copper, U: Uranium, Tl: Thallium.

## Data Availability

Data available on request. The data presented in this study are available on request from the corresponding author.
